# Truncation of *Ube3a-ATS* Unsilences Paternal *Ube3a* and Ameliorates Behavioral Defects in the Angelman Syndrome Mouse Model

**DOI:** 10.1371/journal.pgen.1004039

**Published:** 2013-12-26

**Authors:** Linyan Meng, Richard Erwin Person, Wei Huang, Ping Jun Zhu, Mauro Costa-Mattioli, Arthur L. Beaudet

**Affiliations:** 1 Department of Molecular and Human Genetics, Baylor College of Medicine, Houston, Texas, United States of America; 2 Department of Neuroscience, Baylor College of Medicine, Houston, Texas, United States of America; University of Pennsylvania, United States of America

## Abstract

Angelman syndrome (AS) is a severe neurodevelopmental disorder caused by maternal deficiency of the imprinted gene *UBE3A*. Individuals with AS suffer from intellectual disability, speech impairment, and motor dysfunction. Currently there is no cure for the disease. Here, we evaluated the phenotypic effect of activating the silenced paternal allele of *Ube3a* by depleting its antisense RNA *Ube3a-ATS* in mice. Premature termination of *Ube3a-ATS* by poly(A) cassette insertion activates expression of *Ube3a* from the paternal chromosome, and ameliorates many disease-related symptoms in the AS mouse model, including motor coordination defects, cognitive deficit, and impaired long-term potentiation. Studies on the imprinting mechanism of *Ube3a* revealed a pattern of biallelic transcription initiation with suppressed elongation of paternal *Ube3a*, implicating transcriptional collision between sense and antisense polymerases. These studies demonstrate the feasibility and utility of unsilencing the paternal copy of *Ube3a* via targeting *Ube3a-ATS* as a treatment for Angelman syndrome.

## Author Summary

Angelman syndrome (AS) is a devastating neurodevelopmental disorder diagnosed in young children, currently with no effective treatments. It is characterized by absence of speech, ataxia, intellectual disability, epilepsy, and a characteristic behavior of frequent laughter and smiling. The disease is caused by loss of the maternal allele of *UBE3A*, which is preferentially silenced on the paternal chromosome and expressed on the maternal chromosome in neurons due to genomic imprinting. It has been long proposed that by activating the originally silenced paternal allele of *UBE3A*, the disease may be cured. Here in our research, we demonstrated the feasibility of activating paternal Ube3a in mice by terminating the transcription of its antisense RNA *Ube3a-ATS* genetically. In the AS mouse model who additionally receives the terminated *Ube3a-ATS* allele from the paternal side, we observed restoration of *Ube3a* expression, amelioration of behavioral defects and reversal of the impaired long-term potentiation. We further studied the imprinting mechanisms of *Ube3a* and proposed a novel transcriptional collision model. These results provide solid *in vivo* evidence for a key regulatory role of *Ube3a-ATS* in the disease and open up an exciting possibility of a gene-specific treatment for Angelman syndrome.

## Introduction

Angelman syndrome (AS) is clinically manifested by features of intellectual and developmental disability, absence of speech, ataxic movement, epilepsy, and unique behaviors such as frequent laughter and fascination with water [1], [2]. Despite absence of effective treatment currently, therapeutic development for Angelman syndrome could be potentially optimistic, since patients with AS have overall normal development and brain architecture early in life.

Genetically, the disease is caused by deficiency of an E3 ubiquitin ligase termed UBE3A, which participates in many important neuronal functions such as synaptic development, signal transduction, and plasticity [3]. The gene encoding UBE3A is among a handful of human genes that are subject to genomic imprinting. In neuronal cells, it is highly expressed from the maternal allele, but silenced on the paternal allele. Disruption of the maternal allele, through genomic deletion, paternal uniparental disomy, imprinting defects, or point mutations, leads to the absence of UBE3A expression in neuronal tissues and hence Angelman syndrome. Indeed, in all cases of the disorder, at least one copy of paternal *UBE3A* is intact. One could speculate that by correcting the expression level of *UBE3A* via activating the silenced paternal allele, the disease might be treated.

Imprinted genes usually form clusters in the genome and are controlled by the imprinting center (IC). On human chromosome 15q11–q13, paternally expressed genes, including MAGEL2, *NDN*, *SNRPN*, *SNORD115* and *SNORD116*, are critical genes for Prader-Wiili syndrome (PWS) and form an 2-Mb imprinting cluster together with the AS gene *UBE3A*. Although not fully understood, it is generally believed that the PWS/AS region is regulated by a bipartite imprinting center composed of PWS-IC, which activates genes located in its proximity via looping and direct interacting with them, and AS-IC, which suppresses PWS-IC by transcription-mediated DNA methylation [4], [5]. As a result of combined action of both PWS-IC and AS-IC, the paternal and maternal alleles of *NDN* and *SNRPN* show very distinct epigenetic patterns of DNA methylation and histone modifications [6], [7], [8], which define the paternal alleles as transcriptionally active and maternal alleles as transcriptionally silent.

Imprinting of *UBE3A*, however, is not associated with differential DNA methylation at the promoter region [9], [10]. Instead, it is regulated by its antisense RNA, *UBE3A-ATS*, which is expressed from the paternally inherited chromosome in the brain [11], [12]. As part of the large non-coding transcript (*Shng14*) initiated from the *Snrpn* promoter in mice [13], *Ube3a-ATS* expression is always negatively associated with *Ube3a* sense transcript. For example, when the *Snrpn* promoter was deleted, with or without the Prader-Willi syndrome imprinting center (PWS-IC), the *Ube3a-ATS* level was found to be reduced, coupling with significant up-regulation of paternal *Ube3a* [12], [14]. On the other hand, when maternal *Ube3a-ATS* was activated through replacement of the mouse imprinting center (IC) with the human one, or deletion of the putative AS-IC, maternal *Ube3a* was found to be repressed to some extent [15], [16]. Recently, by terminating *Ube3a-ATS* transcription in neuronally differentiated ES cells, we have showed that paternal *Ube3a* can be activated to a comparable level as maternal *Ube3a* [12], suggesting a direct role of *Ube3a-ATS* in suppressing paternal *Ube3a*.

In the present study, we continue evaluating *Ube3a-ATS* as a potential therapeutic target for treating Angelman syndrome. By characterizing a novel mouse model expressing the truncated form of *Ube3a-ATS*, we provide the first *in vivo* evidence that eliminating *Ube3a-ATS* is sufficient to restore Ube3a expression and improve the abnormal behaviors in the AS mouse model. Mechanisms underlying paternal *Ube3a* silencing are also studied, and a hypothesis of transcriptional collision between *Ube3a* and *Ube3a-ATS* is proposed.

## Results

### Truncation of *Ube3a-ATS* unsilences paternal *Ube3a in vivo*

**Figure 1:**
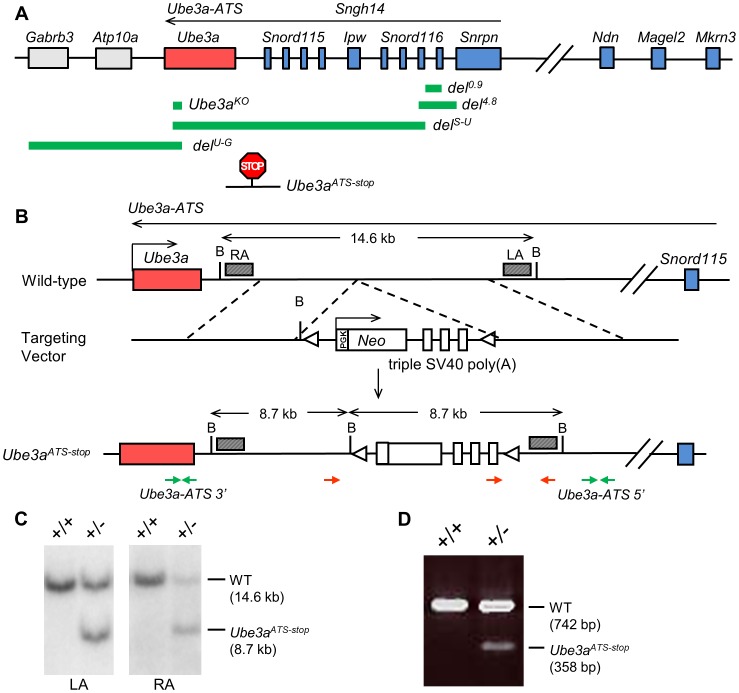
The transcriptional stop cassette was inserted downstream of *Ube3a* via homologous recombination. (**A**) The murine genomic structure of the PWS/AS region is shown (not to scale), with paternally expressed genes in blue, maternally expressed genes in red, and biallelically expressed genes in grey. Genomic regions deleted in the knock-out alleles of *del^0.9^*, *del^4.8^*, *del^S-U^*, *del^U-G^*, and *Ube3a^KO^* are marked by the green bars. Insertion site of the poly(A) cassette in *Ube3a^ATS-stop^* allele is shown by the “STOP” sign. (**B**) The mouse wild-type locus is shown with enlargement of the region between *Snord115* and *Ube3a*. The targeting vector contains the poly(A) cassette and the neomycin selection marker. The slanted boxes are left and right probes used in the Southern blot analysis. LA, left arm; RA, right arm; B, *Bgl*II; red arrows, genotyping primers; green arrows, q-PCR primers of *Ube3a-ATS 5′ and 3′*; triangles, *loxP* sites. (**C**) Recombinant ES cell clones were identified by Southern blot with *Bgl*II digesion. The WT allele gives a band of 14.6 kb and the knock-in allele giving a band of 8.7 kb, hybridizing to either LA or RA probe. (**D**) Chimeric mice were confirmed by multiplex PCR, with WT allele of 742 bp and mutant allele of 358 bp.

In order to test if suppression of *Ube3a-ATS* alone is sufficient to unsilence the paternal allele of *Ube3a*, mice with the *Ube3a^ATS-stop^* allele were generated by inserting the triple SV40 poly(A) cassette [12] in between *Snord115* and *Ube3a* (chr7:66573289 NCBI37/mm9) (Figure 1). This design aims to prevent overlap between *Ube3a* and *Ube3a-ATS* and to minimize its effect on expression of the snoRNA clusters. The inserted cassette also contains a neomycin selection marker in the opposite transcriptional orientation to *Ube3a-ATS* to facilitate and enhance transcriptional termination. The mice were backcrossed to C57/BL6 background for six generations before subsequent expression and behavioral analysis.

**Figure 2:**
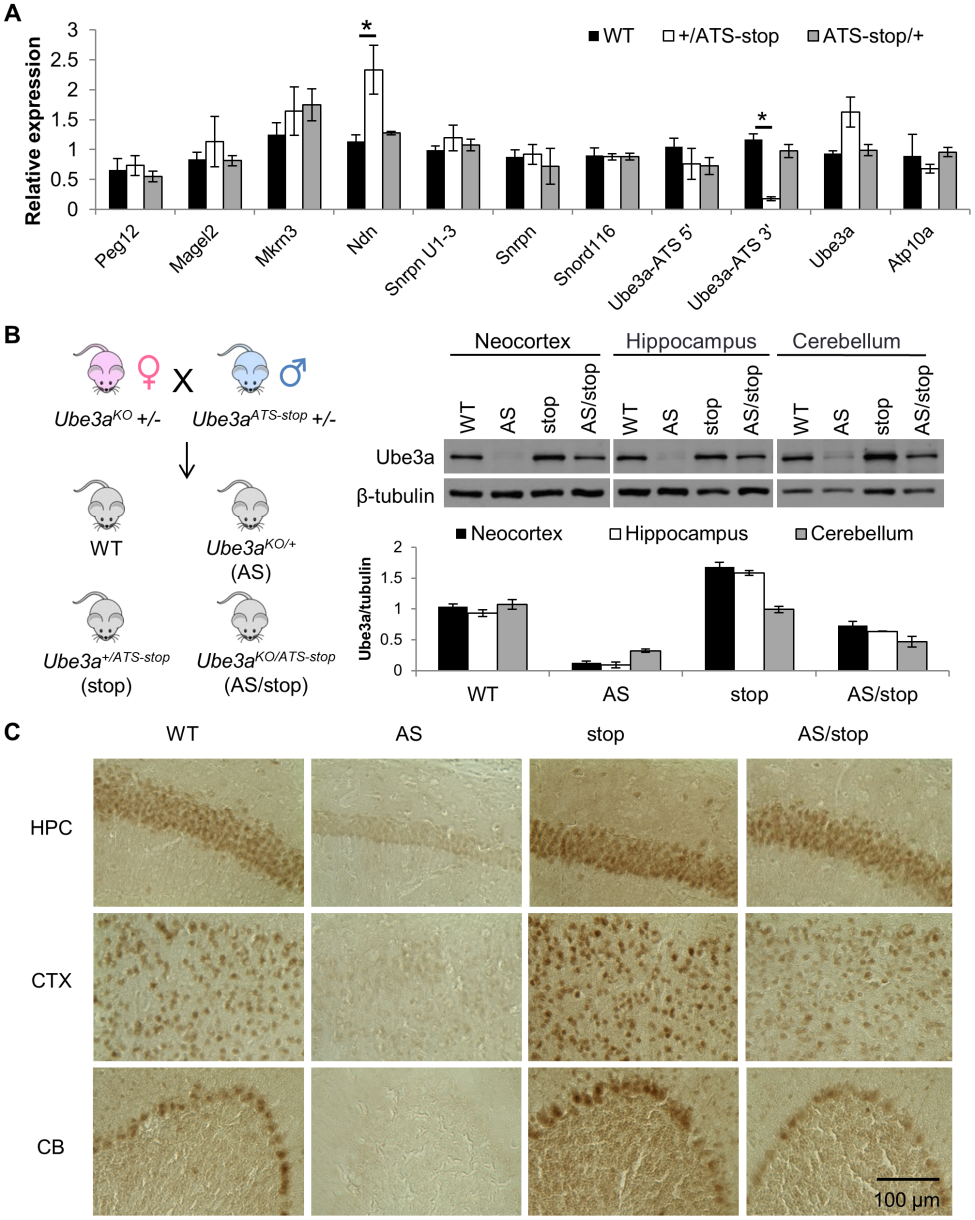
Insertion of the poly(A) cassette into *Ube3a-ATS* leads to its transcriptional termination and unsilencing of paternal *Ube3a*. (**A**) The expression profile of genes in the PWS/AS region was assessed by qRT-PCR in cortices from WT mice and mice inheriting *Ube3a^ATS-stop^* maternally (*ATS-stop/+*) or paternally (*+/ATS-stop*). One set of primers were designed to target *Ube3a-ATS* at loci 5′ and another 3′ to the insertion site. *Gapdh* is used as the internal control. N = 3 per genotype group. Data are averages ± standard error of means (SEM). *p<0.05. (**B**) Male *Ube3a^ATS-stop^* heterozygous mice were bred with female *Ube3a^KO^* heterozygous mice to generate progeny of four different genotypes, WT, *Ube3a^+/ATS-stop^* (stop), *Ube3a^KO/+^* (AS), and *Ube3a^KO/ATS-stop^* (AS/stop). Western blot with anti-Ube3a was performed in neocortex, hippocampus, and cerebellum from these mice. The protein level of Ube3a was normalized to β-tubulin. N = 3 mice per genotype group. Data are averages ± SEM. (**C**) Brain sections from WT, AS, stop, and AS/stop mice were stained with anti-Ube3a. Representative images from CA2 region of hippocampus (HPC), layer II/III of cerebral cortex (CTX), and cerebellum (CB) are shown.

We first determined the effect of the termination cassette on the expression level of *Ube3a-ATS* and other genes located in the imprinting cluster. The *Ube3a-ATS* level downstream of the insertion site (*Ube3a-ATS 3′*, green arrows in Figure 1B) was found to be significantly down-regulated by qPCR analysis when the stop allele was inherited paternally, while maternal inheritance of the allele has no effect (Figure 2A). To exclude the possibility that the PCR amplification site is spliced out instead of terminated, a custom designed strand-specific microarray was further performed as previously reported [12]. A significantly lower level of *Ube3a-ATS* was detected beyond the stop cassette insertion site (Figure S1). Expression of most other imprinted genes located nearby, including *Mkrn3*, *Magel2*, *Snrpn*, *Snord116*, and *Ipw* remained unchanged in both *Ube3a^ATS-stop/+^* and *Ube3a^+/ATS-stop^* mice (maternal genotype precedes the paternal genotype), indicating that the imprinting status of the PWS/AS region is not disrupted by the insertion. The level of *Ndn* was found to be approximately doubled in *Ube3a^+/ATS-stop^* mice compared to the other two genotypes. It is interesting that similar observation has been found in *del^S-U/0.9^* mice previously [12], which expresses *Ube3a-ATS* at a lower level due to *Snrpn* promoter deletion. The reason for the observed up-regulation is unclear.

*Ube3a* mRNA is doubled in the *Ube3a^+/ATS-stop^* mice, suggesting that paternal Ube3a may be unsilenced. To confirm this, male mice heterozygous for *Ube3a-ATS^stop^* were crossed with female mice heterozygous for *Ube3a^KO^* [17] (C57/BL6 background), which is a constitutive *Ube3a* knock-out allele (Figure 1 and 2B). In the progeny, littermates of wild-type (WT), *Ube3a^KO/+^* (AS), *Ube3a^+/ATS-stop^* (stop), *Ube3a^KO/ATS-stop^* (AS/stop) were compared. In the AS/stop mice, Ube3a protein was found to be activated to ∼70% of the WT level in neocortex, ∼60% in hippocampus, and ∼50% in cerebellum. The incomplete activation may be due to leaky termination of *Ube3a-ATS*, as about 20% of *Ube3a-ATS* can still be detected in *Ube3a^+/ATS-stop^* mice (Figure 2A). Immunostaining with anti-Ube3a showed that in AS/stop mice, paternal Ube3a is expressed in most brain regions, including all layers of neocortex, CA1-3 and dentate gyrus of hippocampus, and Purkinje neurons of cerebellum (Figure 2C and S2). Its expression pattern is very similar to that of maternal Ube3a in the WT mice. The incomplete unsilencing of paternal Ube3a may be due to a smaller number of Ube3a positive neurons, or a lower expression level in each single neuron, or more likely a combination of both. Male mice heterozygous for *Ube3a^ATS-stop^* were also crossed with female mice heterozygous for *Ube3a^YFP^* [18], which carries the C-terminal YFP tag (Figure S3). Since Ube3a-YFP is expressed as a fusion protein with a higher molecular weight, it can be easily distinguished from wild-type Ube3a protein by western blot. Inheritance of *Ube3a^ATS-stop^* from the paternal side leads to biallelic expression of Ube3a, while in contrast, maternal inheritance of the allele had no effect.

Finally, the effect of *Ube3a^ATS-stop^* on paternal *Ube3a* was compared with the other two alleles of *del^4.8^* and *del^0.9^*. The allele of *del^4.8^* removes 4.8 kb of *Snrpn* promoter and functions as a PWS-IC deletion, while the allele of *del^0.9^* removes 0.9 kb of *Snrpn* promoter and is equivalent to a *Snrpn* promoter deletion (Figure 1) [19]. After crossing with female mice carrying genomic deletion over the *Snrpn-Ube3a* region (*del^S-U/+^*, Figure 1) [20], the mRNA and protein levels of paternal Ube3a were found to be the highest in *del^S-U/4.8^* mice, intermediate in *del^S-U^/Ube3a^ATS-stop^* mice and the lowest in *del^S-U/0.9^* mice (Figure S4A, B). Interestingly, such order is in accordance with the suppression level of *Ube3a-ATS* (Figure S4C). Plotting of paternal *Ube3a* against *Ube3a-ATS* fits into the curve of exponential decay (R^2^ = 0.997, Figure S4D), suggesting that suppression of paternal *Ube3a* by *Ube3a-ATS* is “dose-dependent”.

### Unsilenced paternal Ube3a ameliorates phenotypic defects in the Angelman syndrome mouse model

We next tested whether inheritance of *Ube3a^ATS-stop^* paternally can correct the phenotypic defects in the Angelman syndrome (AS) mouse model. To address this, male *Ube3a^ATS-stop^* heterozygous mice were crossed with female *Ube3a^KO^* heterozygous mice [17] (C57/BL6 background) and the littermates of WT, *Ube3a^KO/+^* (AS), *Ube3a^+/ATS-stop^* (stop), *Ube3a^KO/ATS-stop^* (AS/stop) were studied for various AS-related phenotypes.

**Figure 3:**
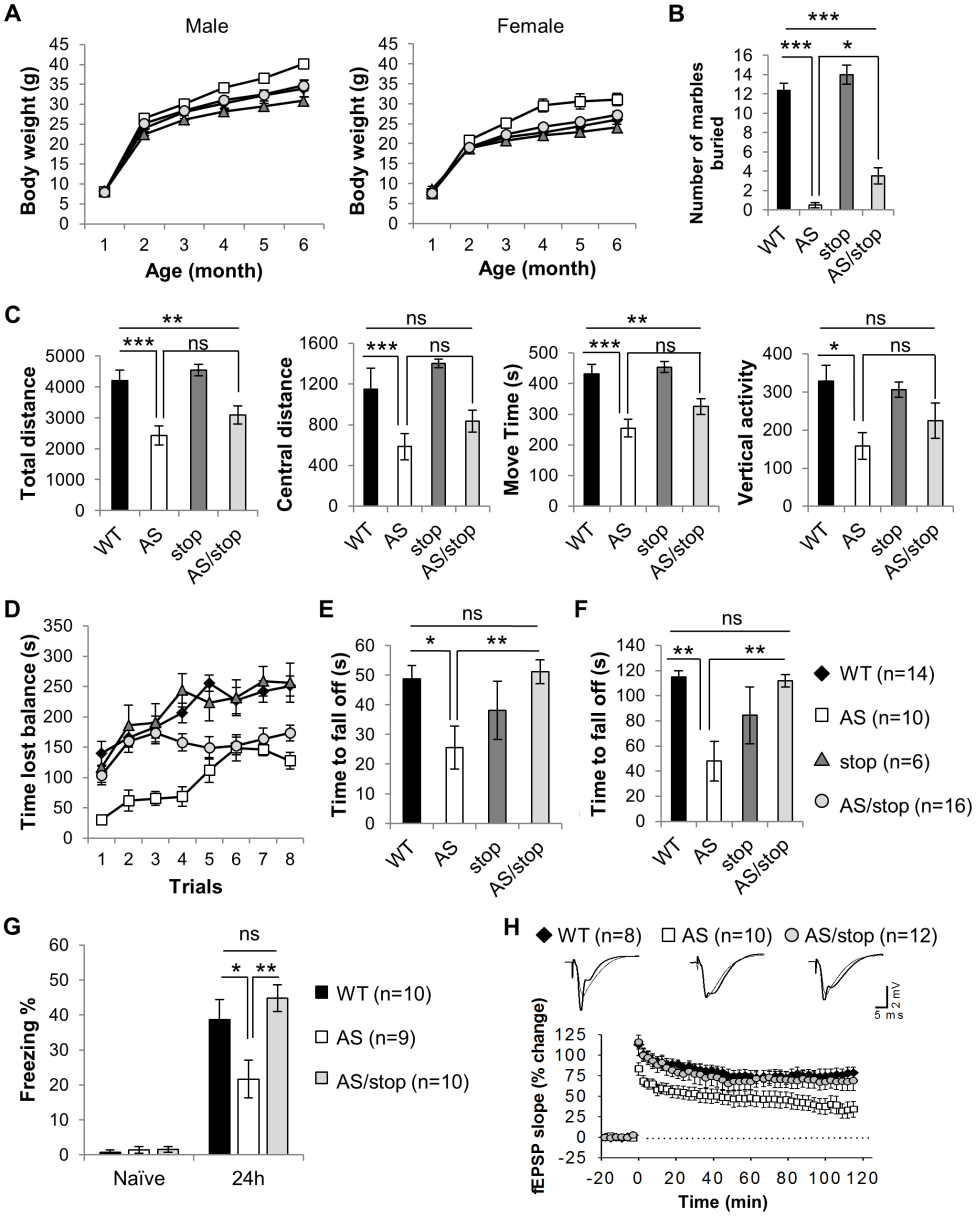
Expression of paternal Ube3a improves phenotypic defects in the AS mouse model. Littermates of WT (black diamonds), AS (white squares), stop (dark gray triangles), and AS/stop (light grey circles) were studied for AS-related phenotypes, including development, behaviors, and electrophysiology. (**A**) Growth curve of male and female mice. (**B**) Marble burying test. (**C**) Open field assay. (**D**) Accelerating rotarod. (**E**) Wire hanging test. (**F**) Dowel test. (**G**) Contextual fear conditioning. (**H**) Long-term potentiation. For the growth curve, the number of animals measured are WT(male) = 9, WT(female) = 10, AS(male) = 9, AS(female) = 5, stop(male) = 7, stop(female) = 5, AS/stop(male) = 9, AS/stop(female) = 11. For the rest experiments, both males and females were tested. Data are averages ± SEM. Error bars in (A) are very tight and are obscured by the symbols. Labels of Black diamonds are overlaid by other symbols. *p<0.05; **p<0.01; ***p<0.001.

Obesity is associated with a small portion of AS patients [2], [9] and constantly observed in many Angelman syndrome mouse models [15], [21], [22]. *Ube3a^KO/+^* mice become overweight starting from three month of age, in both males and females (Figure 3A, p_(WT vs. AS)_<0.01 for 4, 5, 6 months of age, two-way ANOVA of repeated measures). Activation of paternal Ube3a in the AS/stop mice completely reversed the obese phenotype (p_(AS vs. AS/stop)_<0.05 for 4, 5, 6 months of age).

The marble burying test measures repetitive behavior as potentially analogous to an autistic phenotype. Interestingly, AS mice were found to be dramatically impaired in performing this task (Figure 3B, WT: 12.36±0.75, AS: 0.50±0.27, p_(WT vs. AS)_<0.001, one-way ANOVA with Newman-Keuls post-hoc test). AS/stop mice showed a slight but significant improvement over AS mice (AS/stop: 3.50±0.84, p_(AS vs. AS/stop)_<0.05).

Hyperactivity with short attention span is a pronounced problem in young children with AS. Different from humans, AS mice have been reported to display hypoactivity [22], [23]. When placed in an open field and allowed for exploration, AS mice showed significantly lower activity level as measured by total distance and central distance traveled, movement time, and vertical activity (Figure 3C). A slight trend of improvement was consistently observed in the AS/stop mice for these parameters. However, the difference between AS mice and AS/stop mice does not reach statistical significance (one-way ANOVA with Newman-Keuls post-hoc test).

Ataxia and movement difficulty is one of the most severe defects in human AS patients and AS mouse models [17]. AS mice display severe motor coordination defects during the accelerating rotarod test (Figure 3D, p_(WT vs. AS)_<0.05 for all eight trials, two-way ANOVA of repeated measures). AS/stop mice show restoration in the first few trials of accelerating rotarod, although they fail to improve in later trials (p_(AS vs. AS/stop)_<0.05 for trial 1–4). They also show full restoration of other motor defects during wire hanging test and dowel test, indicating a significant improvement of their motor coordination skills (Figure 3E, F, and Figure S5, p_(AS vs. AS/stop)_<0.01 for wire-hanging test and <0.001 for dowel test, one-way ANOVA with Newman-Keuls post-hoc test). It is noted that maternal inheritance of the *Ube3a^ATS-stop^* allele does not affect the performance of the mice in all three motor tests (Figure S6), suggesting that the presence of neomycin cassette has minimal or no effect on motor coordination in mice.

Individuals with AS are frequently affected with specific cognitive deficits [1], [2] and *Ube3a^KO/+^* mice are known to have learning and memory problems [17]. During a fear conditioning test, AS mice exhibited significantly less freezing behavior than did WT littermates (Figure 3G, WT: 38.66±5.78%, AS: 21.68±5.35%, p_(WT vs. AS)_<0.05, one-way ANOVA with Newman-Keuls post-hoc test). Remarkably, the freezing behavior displayed in the AS/stop mice is comparable to the WT mice, suggesting that long-term memory is fully restored (AS/stop: 44.82±3.85%, p_(AS vs. AS/stop)_<0.01).

Lastly, we studied long-term potentiation (LTP) at Schaffer collateral–CA1 synapses, using high-frequency stimulation as the LTP-inducing protocol [17], [24]. As expected, this protocol induced a stable LTP in WT slices but caused a decaying LTP in AS slices (Figure 3H, LTP at 120 min, WT: 78±6.8%, AS: 33±8.6%, p_(WT vs. AS)_<0.01, one-way ANOVA). Notably, the expression of paternal Ube3a reverses the LTP deficits (AS/stop: 63±9.1%, p_(AS vs. AS/stop)_<0.05). The LTP rescue in AS/stop slices cannot be attributed to abnormal basal synaptic transmission, since the relation of fiber volley versus stimulation intensity, initial slope of field EPSPs versus afferent volley size, and paired pulse facilitation were unaltered in these slices (Figure S7).

### Paternal Ube3a is transcriptionally active

**Figure 4:**
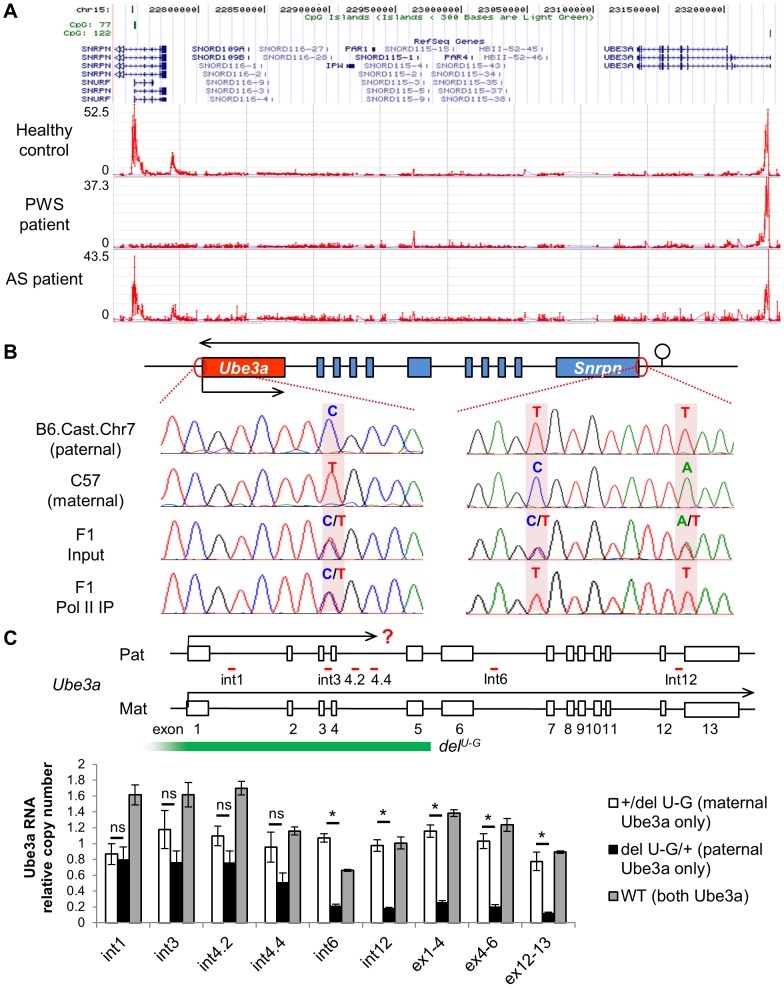
Both promoters of *Ube3a* are enriched with histone H3 lysine 4 trimethylation (H3K4me3), bound by RNA polymerase II, and actively transcribed. (**A**) H3K4me3 of the PWS/AS region was measured in human postmortem cerebellum from healthy control, PWS, and AS patients by ChIP-on-chip. The relative enrichment (signal intensity of IP vs. input) was plotted over the genomic coordinates. (**B**) Binding of RNA polymerase II, a key component of the PIC complex, to the *Ube3a* and *Snrpn* promoters was analyzed by ChIP followed by PCR and sequencing. Cerebral cortices from F1 hybrid of C57 and Cast.Chr7 mice were used for the analysis. SNPs between the two lines were used for allelic identification. (**C**) Relative expression level of paternal versus maternal Ube3a was analyzed in *+/del^U-G^*, *del^U-G^/+*, and WT mice. The copy number of maternal *Ube3a* RNA in *+/del^U-G^* mice was set equal to 1 and the relative expression level of paternal *Ube3a* in *del^U-G^/+* mice or total *Ube3a* in WT mice was calculated. Pre-mRNA of *Ube3a* was measured by strand-specific qRT-PCR using primers located in intronic regions (int1, int3, int4.2, int4.4, int6, and int12), while mature mRNA was measured using primers spanning exon junctions (ex1-4, ex4-6, and ex12-13). Exons of mouse *Ube3a* are displayed as white boxes and deletion region of *del^U-G^* is marked by the green bar. The locations of primers directed to introns are shown with red bars. Pat: paternal; Mat: maternal. N = 3 mice per genotype group. Data are averages ± SEM. *p<0.05.

In developing therapies for treating AS via activating paternal *UBE3A*, it is important to understand the molecular mechanism underlying genomic imprinting of *Ube3a*. Promoters of both the paternal and maternal *UBE3A* remain unmethylated in human brains [9], [10], [25], therefore DNA methylation at the promoter cannot account for silencing of paternal *UBE3A*. In order to look for parent-of-origin epigenetic markers that may account for *UBE3A* imprinting, we first analyzed histone modifications of H3K4 trimethylation (H3K4me3) in human cerebellum tissues by ChIP-on-chip experiment (Figure 4A). In contrast to healthy controls, a PWS patient with a paternal class II deletion (common 4 Mb deletion from break point 2 to 3) lacked H3K4me3 at the *SNRPN* promoter, suggesting that this modification is paternal specific, as previously reported [8]. However, in AS patients with maternal class II deletion, the peak of H3K4me3 was still present at the *UBE3A* promoter, and was indistinguishable from control and PWS samples. Therefore H3K4me3 is equally distributed between the paternal and maternal promoters of *UBE3A* in human cerebellum, regardless of the mono-allelic expression pattern. This conclusion from human was later supported by a recent ChIP-seq study in mice [26], in which equal enrichment of H3K4me3 and H3K27 trimethylation at both parental promoters of *Ube3a* was observed.

We next measured binding of the transcription preinitiation complex (PIC) at the *Ube3a* promoter by chromatin immunoprecipitation (ChIP). The PIC is a large protein complex composed of RNA polymerase II, TATA binding protein (TBP), TFIIB, and many other proteins assembled at the promoter of active genes. ChIP with antibody against RNA polymerase II was performed in brain samples of F1 hybrid of C57 (female) crossed with B6.Cast.Chr7 (male), which carries *Mus. musculus castaneus* chromosome 7 on the *Mus. musculus domesticus* C57BL/6 background. Single nucleotide polymorphisms (SNPs) between the two lines allow detection of parental specific alleles. In contrast to the *Snrpn* promoter, from which only the transcriptionally active paternal allele was precipitated, both parental alleles of the *Ube3a* promoter can be detected in the same IP fraction (Figure 4B). ChIP in F1 hybrids of the reciprocal cross and ChIP with anti-TFIIB and anti-TBP revealed the same result (Figure S8). Altogether, the results suggest that the PIC is able to be properly assembled at the promoter of both paternal and maternal *Ube3a* alleles, regardless of theirs different expression status.

Since paternal *Ube3a* shows multiple features of an active gene as we demonstrated above, we considered the hypothesis that it is actually transcriptionally active despite the absence of mature mRNA. To test this, a mouse model carrying a deletion from *Ube3a* to *Gabrb3* (*del^U-G^*, Figure 1A and 4C) was used [27]. Since the deletion covers the promoter of *Ube3a*, only maternal *Ube3a* RNA is present in the paternal deletion *+/del^U-G^* mice and only paternal *Ube3a* RNA is present in the maternal deletion *del^U-G^/+* mice. We set the RNA copy number of maternal *Ube3a* in *+/del^U-G^* mice equal to 1 across different portions of the gene and used it as the reference to calculate the relative RNA copy number of paternal *Ube3a* in *del^U-G^/+* mice or total *Ube3a* in WT mice. Consistent with the known mono-allelic expression pattern, the mature mRNA of paternal *Ube3a* (quantified by qPCR using primers spanning exon-exon junction) is about 0.2 copy at both the 5′- and 3′-portions in *del^U-G^/+* mice (Figure 4C, ex1-4, ex4-6, and ex12-13). However, when pre-mRNA of paternal *Ube3a* was quantified (by strand-specific qRT-PCR [28] using tagged primers directed to introns), it is around one copy at the 5′ portion of *Ube3a* (black bars of int1, int3, and int4.2 in Fig. 4C) and drops to about 0.2 copy as the primers are moved to the 3′ portion of *Ube3a* (black bars of int4.4, int6, and int12 in Fig. 4C). Altogether, our data supports a model that paternal *Ube3a* is transcribed at a comparable level as maternal *Ube3a* from the promoter, but later becomes suppressed during the process of transcription elongation.

### *Ube3a-ATS* was localized to its transcription site

**Figure 5:**
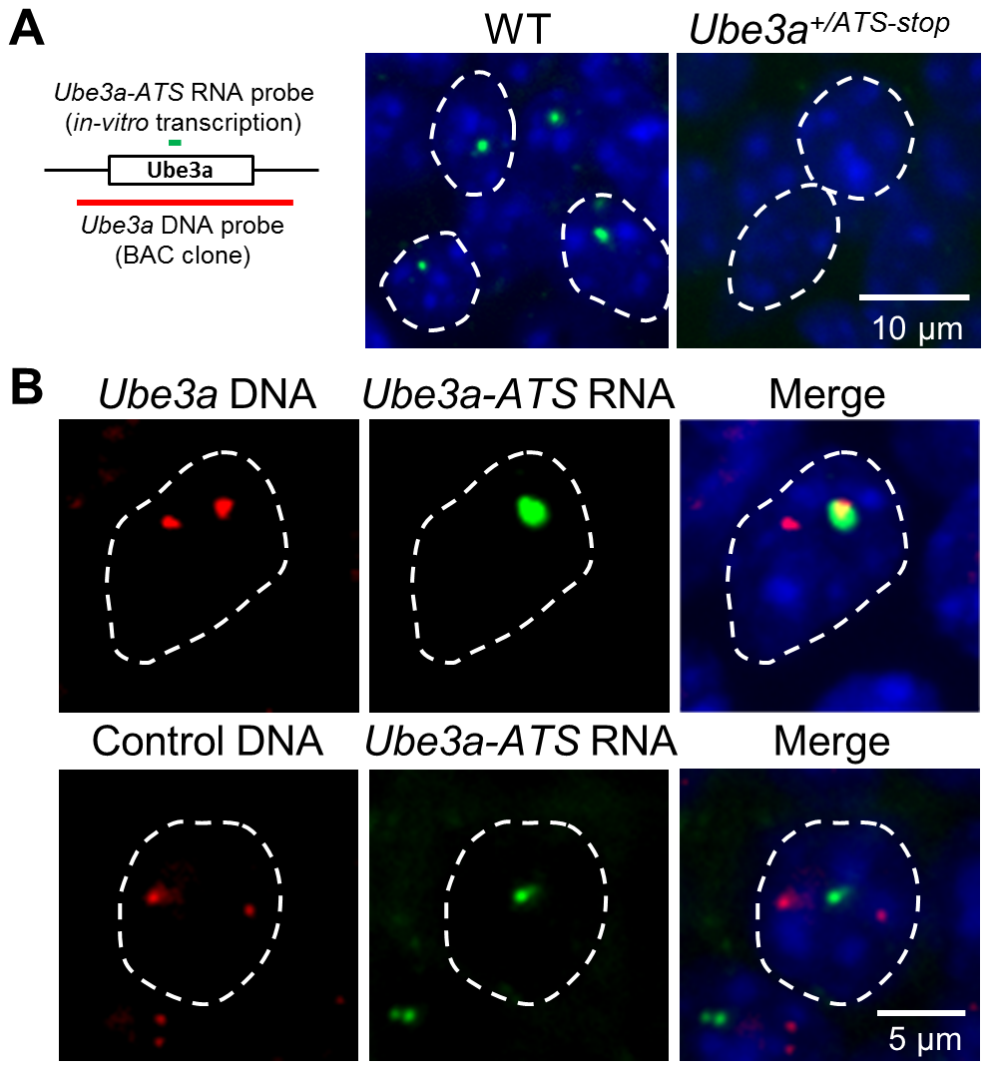
*Ube3a-ATS* localizes to its transcription site in the nucleus. (**A**) Localization of *Ube3a-ATS* RNA was detected by FISH analysis (probe position indicated by the green bar). The signal forms a single dot in the nucleus and is absent in the *Ube3a^+/ATS-stop^* mice, indicating the specificity of the analysis. (**B**) With combined RNA/DNA FISH analysis, signal of *Ube3a-ATS* RNA was found to be co-localized with one of the two foci formed by *Ube3a* DNA (probe position indicated by the red bar, from chromosome 7qC), but not by control DNA targeting irrelevant gene on chromosome 4qD. White dashed lines indicate the boundaries of the nuclei.

*Airn* and *Kcnq1ot1*, two antisense RNAs playing a regulatory role in their respective imprinting cluster, are known based on FISH analysis to be localized around the transcribed regions [29], [30], consistent with their functional roles. *Ube3a-ATS* has been shown to be localized exclusively to the nucleus [12], [31], but the subnuclear detail was unknown. To address this question, a combined RNA/DNA FISH was performed in mouse brain sections. Signals of *Ube3a-ATS* form a single bright dot inside the nucleus (Figure 5A) and can be observed in multiple regions throughout the brain including olfactory bulb, neocortex, hippocampus, cerebellum, and hindbrain. Interestingly, the signal co-localizes with only one of the two foci formed by *Ube3a* DNA signal (Figure 5B). Such co-localization is not random overlapping between the DNA and RNA probes because *Ube3a-ATS* does not overlap with the control DNA probe (targeting an irrelevant gene on mouse chromosome 4). Therefore, similar to *Airn* and *Kcnq1ot1*, *Ube3a-ATS* remains located proximate to its transcription site after it being synthesized.

## Discussion

Patients with Angelman syndrome suffer from developmental delay, speech impairment, and epilepsy. Therapies for AS are limited and focus mainly on symptomatic management [2]. Recently, topoisomerase inhibitors have been identified as the first compounds to successfully unsilence paternal *Ube3a* in mice [32], [33]. In the current research, we investigated a potential therapeutic strategy by activation of the silenced paternal allele of *UBE3A* via suppressing its antisense RNA. Previous studies have defined *Ube3a-ATS* as the negative regulator of *Ube3a* imprinting [12], [14]. However, it was unknown if depletion of *Ube3a-ATS* without modulating other epigenetic factors is sufficient to activate paternal *Ube3a*. This question is crucial in determining whether knock-down of *Ube3a-ATS* is a suitable strategy for treating AS. By generating a mouse model with *Ube3a-ATS* being prematurely terminated, we observed unsilencing of *Ube3a* in multiple brain regions, implying that the antisense RNA plays a regulatory role in modulating *Ube3a* imprinting. We then compared mice which express paternal Ube3a on the maternal Ube3a knock-out background (AS/stop) with AS and WT mice. The AS/stop mice exhibit complete reversal of obesity, motor tests of wire-hanging and dowel walking, fear conditioning defect, and plasticity-related electrophysiology. They also display slight but significant improvement in the tests of accelerating rotarod and marble burying. Therefore, our research confirmed the clinical benefit of activating paternal Ube3a in treating Angelman syndrome and provided a mouse model as the positive control for future drug testing. Given the conservation of the PWS/AS region between mouse and human, activation of paternal *UBE3A* through inhibiting *UBE3A-ATS* expression/transcription should be a promising strategy for developing AS therapy.

One important question in activating paternal *UBE3A* is how much UBE3A protein is needed to achieve phenotypic improvement in AS patients. In the mouse model of AS/stop, we observed some phenotypic reversal, such as obesity, and cognitive deficits. However, their performance during accelerating rotarod and marble bury test is only partially or moderately improved and their decreased locomotive activity is not restored. This may be due to the incomplete activation of paternal Ube3a, which is quantified to be 50–70% of the WT level in different parts of the brain by western blot. Some of the behavioral phenotypes might be more sensitive to the protein level of Ube3a and therefore are more difficult to reverse. Another possibility is the interference from the remaining neomycin cassette. However, paternal inheritance of the cassette on the WT background does not affect mouse behaviors, and maternal inheritance of the cassette does not change Ube3a expression and rotarod performance in mice, suggesting that the presence of the selection marker has no or minimal effect on Ube3a function. Among the human *UBE3A* mutations that have been reported so far, there is a striking preponderance of frameshift and nonsense mutations [34]. It is possible that individuals with less pathogenic missense mutation in *UBE3A* display some, but not all, clinical features associated with AS and thus are excluded from AS diagnosis and research. A patient with C21Y missense mutation located outside the HECT domain of *UBE3A* has been reported to have a less classical phenotype [35], suggesting that partial activity of *UBE3A* may be beneficial.

Another relevant issue is to understand the molecular mechanism underlying *UBE3A* imprinting. Interestingly, several pieces of evidence have suggested that the paternal allele of *UBE3A/Ube3a* is transcriptionally active. For example, the promoter of paternal *UBE3A* is unmethylated [9], [10], [36], modified with active histone markers (Figure 4A), and bound with transcription pre-initiation factors (Figure 4B). Indeed, *Ube3a* pre-mRNA can be detected equally from the 5′-portion of both paternal and maternal alleles in mice (Figure 4C). Therefore, paternal Ube3a is transcriptionally active and its suppression may occur during the process of transcription elongation. The previous observation of “biallelic” expression pattern at the 5′-portion of mouse *Ube3a* by SNP analysis is consistent with this conclusion [37].

As demonstrated in this and many other studies, *Ube3a-ATS* has a direct role in silencing paternal *Ube3a*. However the detailed mechanism is unclear. Research on the other two imprinted ncRNA *Airn* and *Kcnq1ot1* has raised two different working models, promoter occlusion and RNA-directed targeting. When silencing the overlapping gene in embryonic tissues, *Airn* transcribes through the *Igf2r* promoter and precludes binding of RNA polymerase II to the *Igf2r* promoter [38]. In contrast, when silencing the respective non-overlapping genes in extraembryonic tissues, the RNA product of *Kcnq1ot1* or *Airn* will bind to trans-acting protein factors and induce repressive higher-order chromatin changes [29], [39], [40], [41]. Can either of the two models be applied to *Ube3a-ATS*? Promoter occlusion is unlikely to be the cause of *Ube3a* imprinting since paternal *Ube3a* promoter is transcriptionally active. Components of PIC such as RNA polymerase II, TBP, and TFIIB are found to bind paternal and maternal *Ube3a* equally. Currently, it is unknown whether the RNA product of *Ube3a-ATS* is essential in mediating *Ube3a* imprinting. However, *Ube3a-ATS* has very low homology between mouse and human, and is quickly degraded [12], implying a low functional importance of the RNA product.

**Figure 6:**
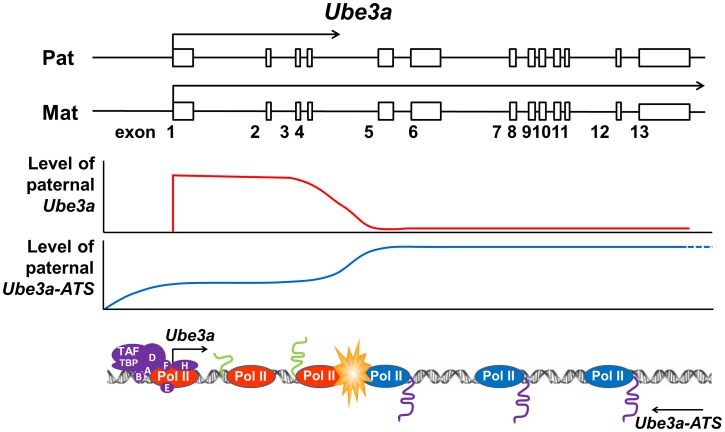
Transcriptional collision hypothesis of *Ube3a* imprinting. Paternal *Ube3a* was found to be transcribed at a similar level as maternal *Ube3a* at the 5′ portion of the gene. The levels of both paternal *Ube3a* sense and antisense transcripts were found to be decreased around intron 4 of *Ube3a*. Based on these observations, a hypothesis of transcriptional collision is proposed in which the two opposing polymerases of *Ube3a* and *Ube3a-ATS* on the paternal chromosome collide into each other around intron 4 of *Ube3a*. The collision causes the polymerases to stall or fall off from the templates, thereby completely or partially terminating the transcription of *Ube3a* and *Ube3a-ATS*. The incomplete sense transcript of *Ube3a* is then degraded and unable to be processed into full-length mature mRNA.

Here we proposed an alternative hypothesis of transcriptional collision as the mechanism for *Ube3a-ATS* mediated Ube3a imprinting (Figure 6). Our previous strand-specific microarray data revealed a significant decrease of *Ube3a-ATS* RNA signal around intron 4 of *Ube3a*, although the transcript remains detectable until ∼40 kb upstream of *Ube3a* promoter [12]. Interestingly, this is around the same region where the pre-mRNA level of paternal *Ube3a* becomes suppressed. Therefore, on the paternal chromosome, *Ube3a* sense and antisense RNAs are transcribed head-to-head at a relatively high level until the polymerases reach intron 4, where both drop to a lower level. These findings are similar to what has been described for transcriptional collision occurring during convergent transcription [42]. Such collision will result in stalling, dissociation of both polymerases, and abortive transcription of both. Research in budding yeast has demonstrated both *in vitro* and *in vivo* that convergent transcription will result in collision of the two opposing polymerases [42], [43]. The collision event can also be detected by atomic force microscopy *in vitro* when two promoters are aligned convergently on a linear DNA template [44]. Currently, we still lack direct evidence to support the *Ube3a* transcriptional collision hypothesis. It will be necessary to test it in the future by mapping RNA polymerase II stalling sites along *Ube3a* using GRO-seq or NET-seq technology [45], [46].

## Materials and Methods

### Ethics statement

All animal procedures were performed in accordance with NIH guidelines and approved by the Baylor College of Medicine Institutional Animal Care and Use Committee (IACUC). All human studies were performed in accordance with NIH guidelines and approved by the Baylor College of Medicine Institutional Review Board (IRB).

### Generation of *Ube3a^ATS-stop^* mice

The insertion cassette composed of SV40 triple poly(A) signal and neomycin selection marker was inserted downstream of *Ube3a* by gene targeting in wild-type AB2.2 ES cells. After microinjecting into blastocysts of C57/BL6 mice, high percentage male agouti chimeras were obtained and germline transmission was established. The lines were then backcrossed to C57/BL6 mice for more than six generations. PCR genotyping was developed with TS-F (TTCCCAGTGCTGAGACTAAAG), TS-R (CCACAATCTGAA-CCCTAAAAC) and SV40-R (AAAAGGGACAGGATAAGTATG).

### RNA extraction and qRT-PCR

Total RNA was prepared with miRNeasy Mini Kit (Qiagen, Valencia, CA). On-column DNase treatment was performed for all the samples. The cDNA was generated using 0.2–1 µg of total RNA with SuperScript III First-Strand Synthesis System (Invitrogen, Carlsbad, CA), and qRT-PCR was performed using Applied Biosystems StepOnePlus Real-Time PCR System and SYBR Green Master Mix (Applied Biosystems, Carlsbad, CA). Primers used are listed in Table S1.

### Western blot

Western blot against Ube3a and β-tubulin was performed as previously described [12]. Quantification was performed based on densitometry with ImageJ.

### Histology and immunohistochemistry

Tissue preparation and immunohistochemistry were performed by Neuropathology Core of Baylor College of Medicine, as previously described [47]. Immunostaining was carried out with Rabbit polyclonal anti-Ube3a (1∶500, A300-352A, Bethyl Laboratories, Montgomery, TX) and horseradish peroxidase conjugated goat anti-rabbit (1∶200, Dako Inc., Carpinteria, CA). The localization of the antibody was visualized using diaminobenzidine (DAB, 0.5 mg/ml, Vector Laboratories Inc., Burlingame, CA) as a chromogen.

### Behavioral tests

A battery of behavioral tests was performed using a protocol previously described and used in Behavioral Core facilities at Baylor College of Medicine [27], [48]. A detailed protocol for each test is described in Text S1. Tests start when the mice are 2 month-old in both males and females and the order of tests are kept the same as listed in the supplementary material. The interval between two tests is one week, except wire hanging, dowel tests and rotarod were performed in two consecutive days.

### Electrophysiology

Horizontal hippocampal slices (350 µm) were cut with a Leica (VT 1000S) vibratome (Buffalo Grove, IL) from brains of WT, AS and AS/stop mice in 4°C artificial cerebrospinal fluid (ACSF) and kept in ACSF at room temperature for at least one hour before recording, as previously described [49], [50]. Slices were maintained in an interface-type chamber perfused (2–3 ml/min) with oxygenated ACSF (95% O_2_ and 5% CO_2_) containing in mM: 124 NaCl, 2.0 KCl, 1.3 MgSO_4_, 2.5 CaCl_2_, 1.2 KH_2_PO_4_, 25 NaHCO_3_, and 10 glucose. Bipolar stimulating electrodes were placed in the CA1 stratum radiatum to excite Schaffer collateral and commissural fibers. Field EPSPs were recorded at 30–31°C, with ACSF-filled micropipettes. The recording electrodes were placed in the stratum radiatum and the intensity of the 0.1 ms pulses was adjusted to evoke 40–50% of maximal response. A stable baseline of responses at 0.033 Hz was established for at least 20 min. Tetanic LTP was induced by using two 1 s, 100 Hz tetani, 20 s apart at baseline stimulus intensity, as previously described [17].

### Human postmortem brain samples

Postmortem brain tissues from control, PWS, and AS individuals were obtained from NICHD Brain and Tissue Bank for Developmental Disorders from University of Maryland School of Medicine.

### Chromatin immunoprecipitation and microarray analysis of human tissues

ChIP-on-chip analysis was performed as previously described [51]. Immunoprecipitation was performed with Protein A Dynabeads (Invitrogen) coated with normal rabbit IgG or anti-H3K4me3 antibodies (17-614, Millipore) according to manufacturer's instructions. Precipitated and input DNA was amplified with GenomePlex Complete Whole Genome Amplification (WGA) kit (Sigma, St. Louis, MO) and labeled with Cy3 (input DNA) or Cy5 (ChIP DNA) using BioPrime Array CGH Genomic Labeling System (Invitrogen). The DNA was then applied to a custom designed human chromosome 15q11.2–q12 focused array (Agilent, Santa Clara, CA), with genomic tilling probes covering regions from *MAGEL2* to *GABRB3* (chr15:21,361,151–25,487,147, genome build NCBI36/hg18) in the 4X44k format. Hybridization, wash and scanning were performed according to manufacturer's instructions. The image files were processed with Agilent Feature Extraction software using protocol CGH-v4_95_Feb07 and further analyzed with Agilent G4477AA ChIP Analytics 1.3 software.

### ChIP-PCR and SNP identification

Brain tissues of 50 mg from newborn mice was chopped into fine pieces, crosslinked with 1% formaldehyde in DMEM and lysed in SDS lysis buffer (50 mM Tris, 10 mM EDTA, 1% SDS). The lysate was then sonicated (Fisher Scientific 500 Sonic Dismembrator) and centrifuged. The supernatant was collected and combined with IP buffer (2 mM Tris, 15 mM NaCl, 0.2 mM EDTA, 0.1% Triton X-100, 1× proteinase inhibitor). Immunoprecipitation was then performed with Protein G Dynabeads (Invitrogen) coated with anti-pol II (05-623, Millipore), anti-TFIIB (sc-225, Santa Cruz Biotechnology), or anti-TBP (MAB3658, Millipore) overnight.

Immunoprecipitated DNA was PCR amplified with *Snrpn* or *Ube3a* promoter primers, purified with MinElute PCR purification kit (Qiagen) and analyzed by Sanger sequencing to identify allelic SNPs. Alternatively, unpurified PCR products were digested with *Bsa*I for the *Snrpn* promoter or *Bbs*I for the *Ube3a* promoter, and analyzed by eletrophoresis on a 1.5% agarose gel.

### Strand-specific qRT-PCR

Total RNA of 200 ng from cortices of newborn mice was used as the input in the analysis. The cDNA synthesis was performed using tagged gene-specific primers in the RT reaction to detect *Ube3a* pre-mRNA in a strand-specific manner [28], and then amplified in the SYBR Green q-PCR system using the tag as the reverse primer and locus specific forward primer. All primers used are listed in Table S2.

### DNA/RNA combined fluorescent in-situ hybridization (FISH)

Tissue preparation and RNA FISH were carried out by RNA In-Situ Hybridization Core at Baylor College of Medicine as previously described [52]. Briefly, brains of adult mice were embedded in O.C.T., fresh frozen, and sectioned sagittally at 25 µm thickness. After paraformaldehyde fixation, acetylation, and dehydration, the slides were assembled into flow-through hybridization chambers and placed into a Tecan (Mannedorf, Switzerland) Genesis 200 liquid-handling robot. The DIG labeled RNA probes were prepared with *in-vitro* transcription and corresponded to chr7:66,530,657–66,531,391(NCBI37/mm9). Primers for DNA template synthesis are SP6-Ube3a-int5.1F **ATTTAGGTGACACTATAGAAGCG**AAGATGAGTCAG-TTTGGTTTT and T7-Ube3a-ex6.1R **TAATACGACTCACTATAGGGAGA**TTCTGAGTCTTCTTCCATA-GC). The T7 promoter was used to generate *Ube3a-ATS* probe. Hybridized probes were detected by a dual amplification strategy and visualized by Alexa488 conjugated streptavidin [52].

After RNA FISH, the slides were washed in 2XSSC at 37°C for 15 min, dehydrated in 70%, 85%, 95% ethanol at −20C for 2 min each and denatured in 70% formamide/2XSSC at 70°C for 2 min. After washing with 70%, 85% and 100% ethanol, the slides were air-dried before hybridization with the DNA FISH probe. The probe was prepared from Ube3a BAC clone bMQ311i10 (Source BioScience, UK) or Lepre1 (chr 4) with FISH Tag DNA Red Kit (Invitrogen) and hybridized to the sections at 37°C overnight. The slides were washed in 50% formamide/2X SSC solution twice for 8 min at 42°C, once in 2XSSC for 8 min at 37°C and mounted with SlowFade Gold antifade reagent (Invitrogen).

### Statistical analysis

Statistical analysis was performed with GraphPad Prism 5 (GraphPad Software, Inc. La Jolla, CA). One-way ANOVA with Newman–Keuls post-hoc test and two-way ANOVA with repeated measures were used.

## Supporting Information

Figure S1 Insertion of poly(A) cassette on the paternal chromosome in *Ube3a^+/ATS-stop^* mice terminates the transcription of *Ube3a-ATS*. Total RNAs prepared from cerebrum of WT and *Ube3a^+/ATS-stop^* mice are subject to custom designed strand-specific microarray analysis. The normalized signal intensity is plotted over genomic coordinates (NCBI37/mm9 build), with the moving average of 10 probes.
(TIF)

Figure S2 Paternal Ube3a is unsilenced in AS/stop mice. Brain sections from adult WT, AS, stop, and AS/stop mice were analyzed by immunohistochemistry with anti-Ube3a. Shown here in the figures are hippocampus (**A**) and cerebral cortex (**B**).
(TIF)

Figure S3 Paternal inheritance of *Ube3a^ATS-stop^* leads to biallelic expression of Ube3a, while maternal inheritance of the allele has no effect on Ube3a expression. (**A**) Male *Ube3a^ATS-stop^* heterozygous mouse was crossed with female *Ube3a^YFP^* heterozygous mice to generate progeny of *Ube3a^YFP/+^* and *Ube3a^YFP/ATS-stop^* mice. The reciprocal cross was also carried out. (**B**) The scheme shows the two alleles of *Ube3a^ATS-stop^* and *Ube3a^YFP^*. *Ube3a^YFP^* carries a C-terminal YFP tag, which leads to expression of the Ube3a-YFP fusion protein with a higher molecular weight. (**C**) Western blot with anti-Ube3a was performed with cerebrum from *Ube3a^YFP/+^*, *Ube3a^YFP/ATS-stop^*, *Ube3a^+/YFP^*, and *Ube3a^ATS-stop/YFP^* mice. β-tubulin is used as the loading control. (D) The amount of Ube3a-YFP and Ube3a protein normalized to β-tubulin was quantified in four groups of mice. (E) The ratio of paternal to maternal Ube3a was calculated and plotted. Pat: paternal; Mat: maternal. Data are averages ± range.
(TIF)

Figure S4 The effect of paternally inherited *Ube3a^ATS-stop^* allele on activating cis *Ube3a* is compared with *del^0.9^* and *del^4.8^*. (**A**) RNA levels of *Ube3a* were quantified by qRT-PCR in cerebrum of newborn mice and compared among different genotypes. It is normalized to the internal control of *Pgk1*. (**B**) Protein levels of Ube3a was measured by western blot and normalized to β-tubulin. (**C**) *Ube3a-ATS* RNA was analyzed by qRT-PCR. (**D**) RNA levels of paternal *Ube3a* (in *del^S-U/+^*, *del^S-U/0.9^*, *del^S-U^/Ube3a^ATS-stop^*, and *del^s-u/4.8^* mice) were plotted over *Ube3a-ATS*. Non-linear regression of exponential decay was performed with the best fitting curve shown in red (R^2^ = 0.997). Data are averages ± range. N = 3 mice per genotype group.
(TIF)

Figure S5 AS/stop mice display normal behaviors in wire-hanging test and dowel test. A second batch of WT, AS, stop, and AS/stop mice were analyzed for wire-hanging and dowel test. WT = 14, AS = 11, stop = 13, AS/stop = 9. Both male and females are included. Data are averages ± SEM. ***p<0.001, ns: not significant.
(TIF)

Figure S6 Maternal inheritance of the *Ube3a^ATS-stop^* allele does not affect motor performance in mice. (**A**) Accelerating rotarod. (**B**) Wire-hanging test. (**C**) Dowel test. There is no statistical significance between the two groups in these tests.
(TIF)

Figure S7 WT, AS, and AS/Stop mice have similar basal synaptic transmission in CA1 slices. (**A**) Pesynaptic fiber volley as a function of stimulus intensity showed no difference in fiber excitability among WT, AS, and AS/stop mice. The mean afferent volley at a given stimulation strength was fitted by linear regressions. (**B**) Input-output relation of fEPSPs as function of presynaptic fiber volley amplitude over a wide range of stimulus intensities was also similar for WT, AS, and AS/stop slices. (**C**) Paired-pulse facilitation of fEPSPs did not significantly differ between WT, AS and AS/Stop slices, as demonstrated by the plots of the paired-pulse ratio (fEPSP2/fEPSP1) for various intervals of paired stimulation.
(TIF)

Figure S8 The promoters of both paternal and maternal *Ube3a* are bound by TFIIB, a component of the preinitiation complex. Cerebral cortices from F1 hybrid of C57 and Cast.Chr7 mice were used for ChIP analysis against TFIIB. Precipitated DNA was PCR amplified for *Ube3a* and *Snrpn* promoters and subject to restriction enzyme digestion to identify parental alleles. *Snrpn* promoter from C57 but not Cast.Chr7 can be cut by *Bsa*I. *Ube3a* promoter from Cast.Chr7 but not C57 can be cut by *Bbs*I.
(TIF)

Table S1 Q-PCR primers used in the expression analysis.
(DOCX)

Table S2 RT and q-PCR primers used in analyzing *Ube3a* transcription initiation.
(DOCX)

Text S1 Materials and methods.
(DOCX)
