# Supplementary figures and images for: Truncation of Ube3a-ATS Unsilences Paternal Ube3a and Ameliorates Behavioral Defects in the Angelman Syndrome Mouse Model
Source: PLoS Genet. 2013 Dec 26;9(12):e1004039. doi: 10.1371/journal.pgen.1004039 (PMC3873245; doi:10.1371/journal.pgen.1004039)

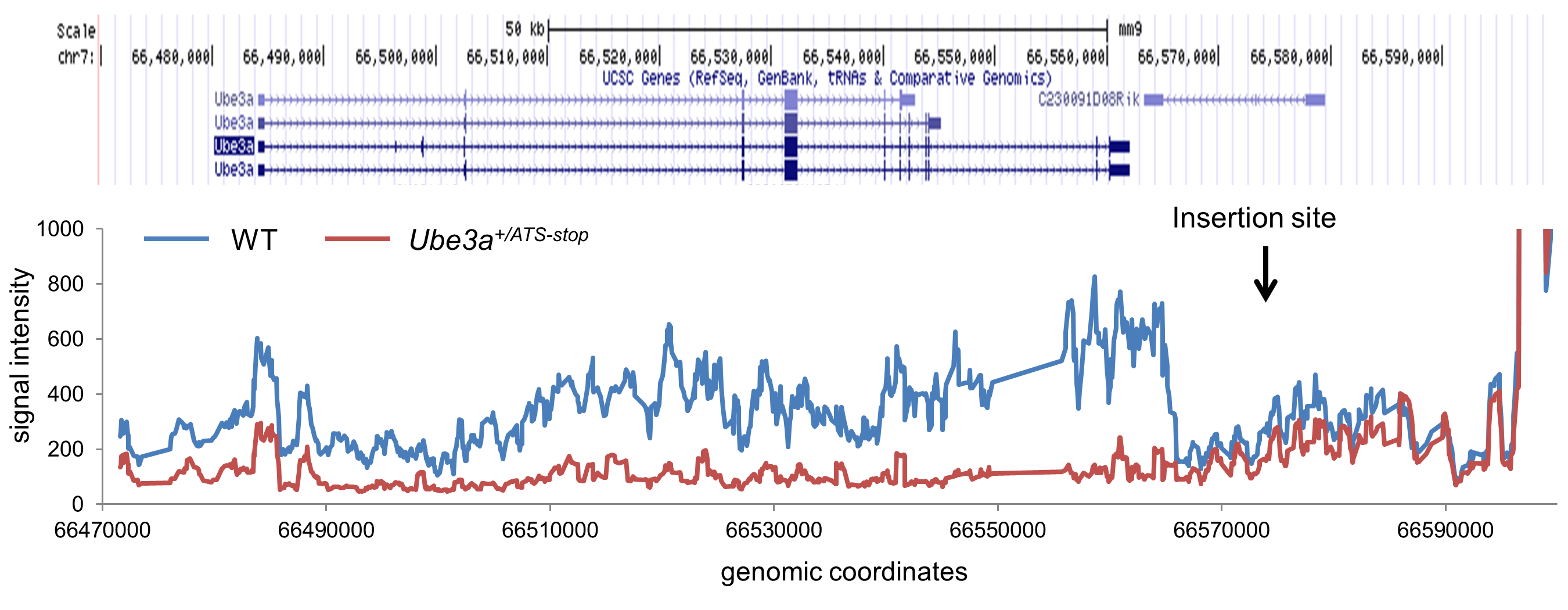

Supplement: Figure S1 — Insertion of poly(A) cassette on the paternal chromosome in Ube3a+/ATS-stop mice terminates the transcription of Ube3a-ATS. Total RNAs prepared from cerebrum of WT and Ube3a+/ATS-stop mice are subject to custom designed strand-specific microarray analysis. The normalized signal intensity is plotted over genomic coordinates (NCBI37/mm9 build), with the moving average of 10 probes. (TIF) [file pgen.1004039.s001.tif]

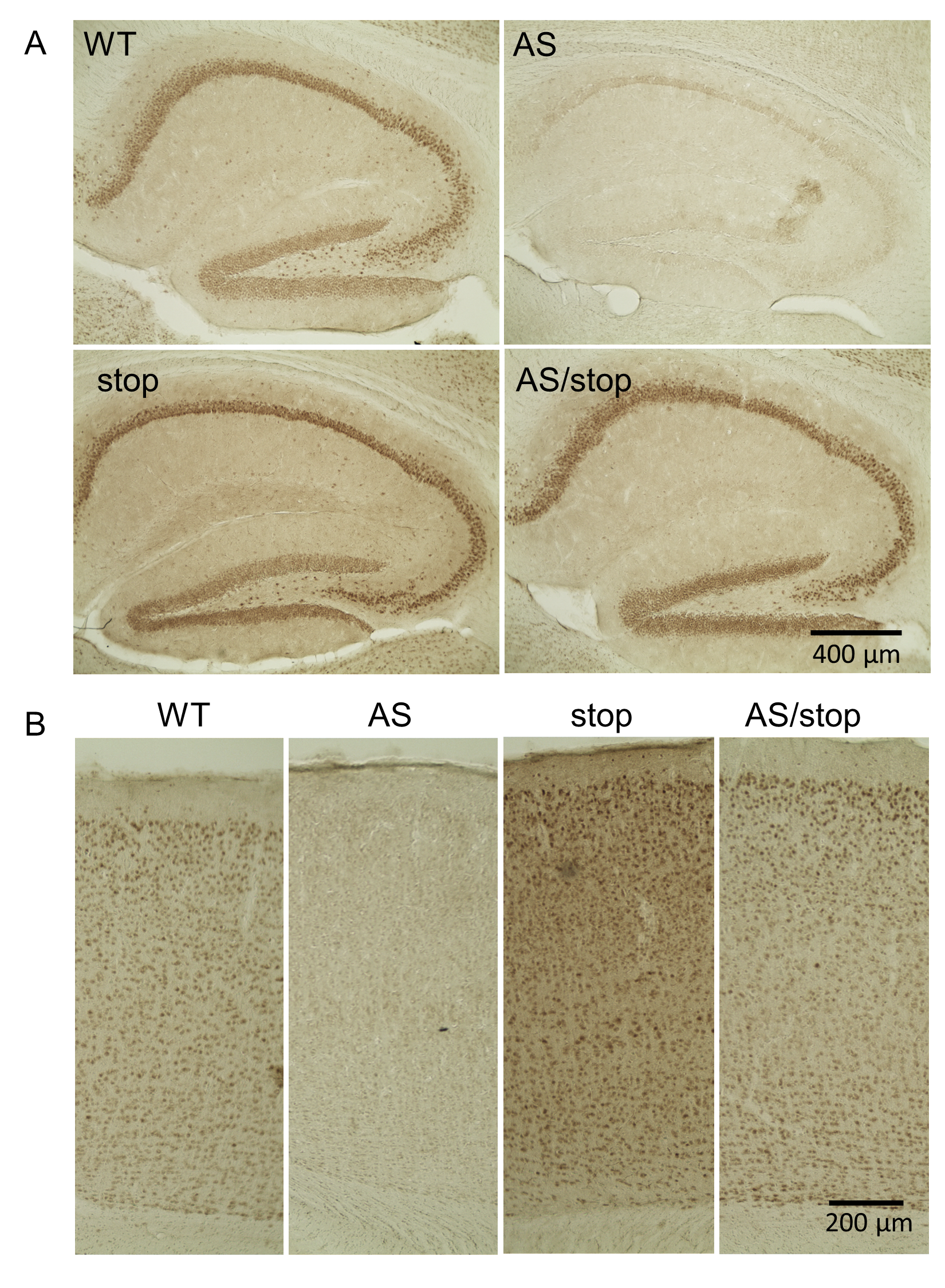

Supplement: Figure S2 — Paternal Ube3a is unsilenced in AS/stop mice. Brain sections from adult WT, AS, stop, and AS/stop mice were analyzed by immunohistochemistry with anti-Ube3a. Shown here in the figures are hippocampus (A) and cerebral cortex (B). (TIF) [file pgen.1004039.s002.tif]

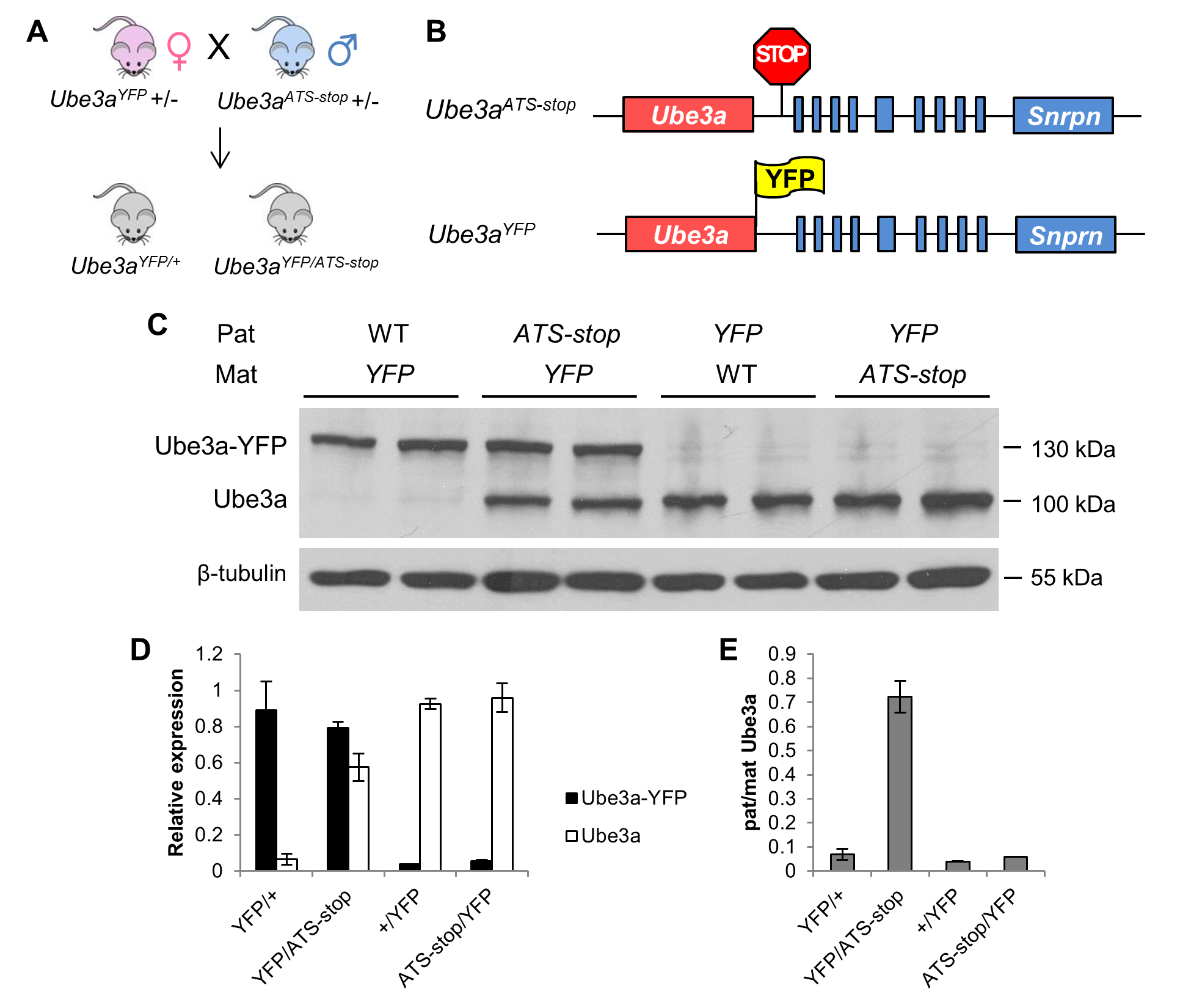

Supplement: Figure S3 — Paternal inheritance of Ube3aATS-stop leads to biallelic expression of Ube3a, while maternal inheritance of the allele has no effect on Ube3a expression. (A) Male Ube3aATS-stop heterozygous mouse was crossed with female Ube3aYFP heterozygous mice to generate progeny of Ube3aYFP/+ and Ube3aYFP/ATS-stop mice. The reciprocal cross was also carried out. (B) The scheme shows the two alleles of Ube3aATS-stop and Ube3aYFP. Ube3aYFP carries a C-terminal YFP tag, which leads to expression of the Ube3a-YFP fusion protein with a higher molecular weight. (C) Western blot with anti-Ube3a was performed with cerebrum from Ube3aYFP/+, Ube3aYFP/ATS-stop, Ube3a+/YFP, and Ube3aATS-stop/YFP mice. β-tubulin is used as the loading control. (D) The amount of Ube3a-YFP and Ube3a protein normalized to β-tubulin was quantified in four groups of mice. (E) The ratio of paternal to maternal Ube3a was calculated and plotted. Pat: paternal; Mat: maternal. Data are averages ± range. (TIF) [file pgen.1004039.s003.tif]

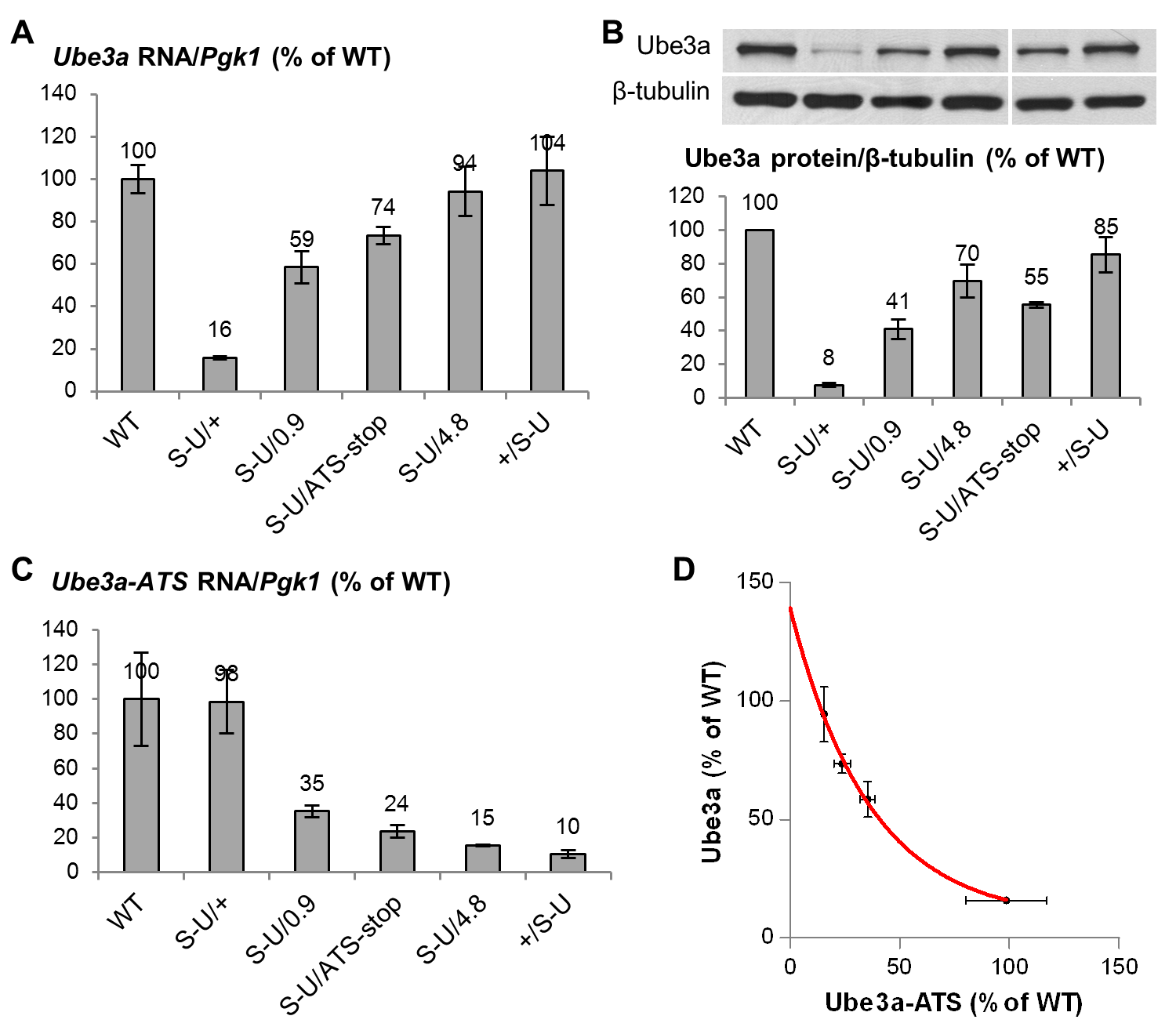

Supplement: Figure S4 — The effect of paternally inherited Ube3aATS-stop allele on activating cis Ube3a is compared with del0.9 and del4.8. (A) RNA levels of Ube3a were quantified by qRT-PCR in cerebrum of newborn mice and compared among different genotypes. It is normalized to the internal control of Pgk1. (B) Protein levels of Ube3a was measured by western blot and normalized to β-tubulin. (C) Ube3a-ATS RNA was analyzed by qRT-PCR. (D) RNA levels of paternal Ube3a (in delS-U/+, delS-U/0.9, delS-U/Ube3aATS-stop, and dels-u/4.8 mice) were plotted over Ube3a-ATS. Non-linear regression of exponential decay was performed with the best fitting curve shown in red (R2 = 0.997). Data are averages ± range. N = 3 mice per genotype group. (TIF) [file pgen.1004039.s004.tif]

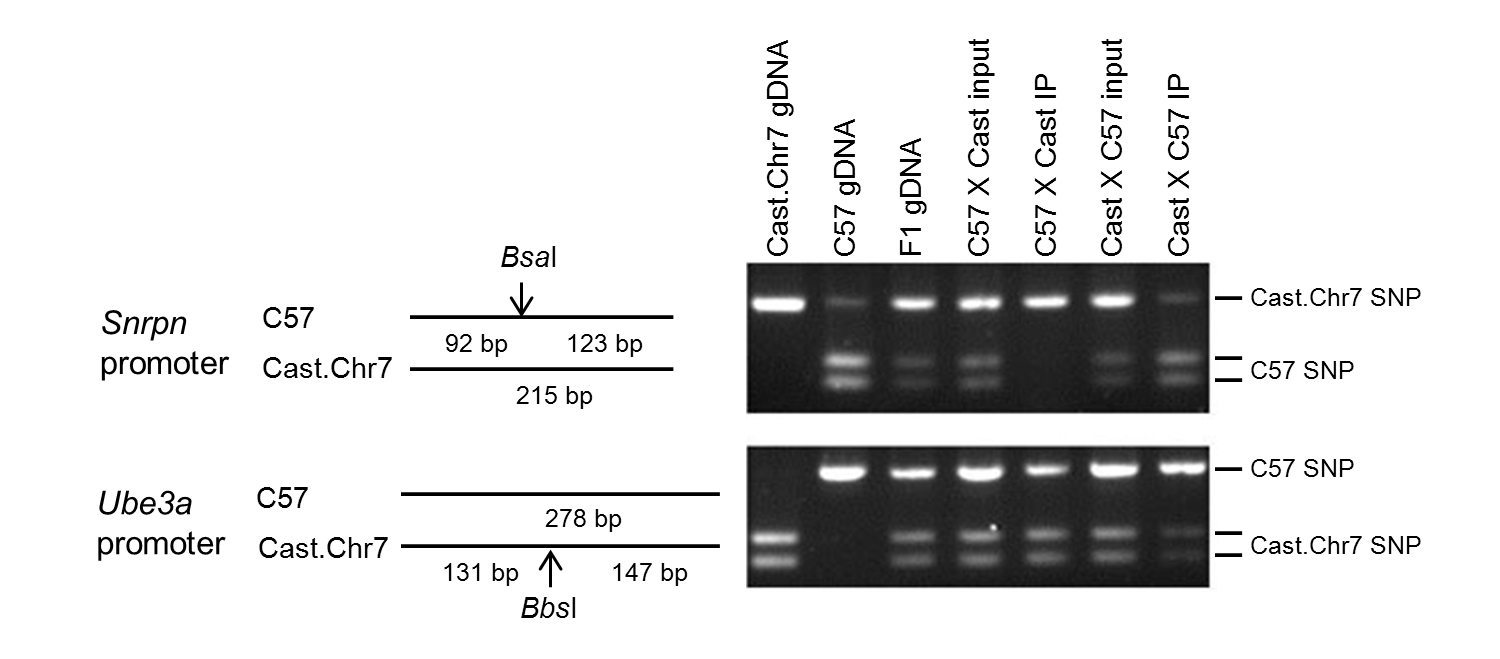

Supplement: Figure S8 — The promoters of both paternal and maternal Ube3a are bound by TFIIB, a component of the preinitiation complex. Cerebral cortices from F1 hybrid of C57 and Cast.Chr7 mice were used for ChIP analysis against TFIIB. Precipitated DNA was PCR amplified for Ube3a and Snrpn promoters and subject to restriction enzyme digestion to identify parental alleles. Snrpn promoter from C57 but not Cast.Chr7 can be cut by BsaI. Ube3a promoter from Cast.Chr7 but not C57 can be cut by BbsI. (TIF) [file pgen.1004039.s008.tif]
