# Supplementary material for: Truncation of Ube3a-ATS Unsilences Paternal Ube3a and Ameliorates Behavioral Defects in the Angelman Syndrome Mouse Model
Source: PLoS Genet. 2013 Dec 26;9(12):e1004039. doi: 10.1371/journal.pgen.1004039 (PMC3873245; doi:10.1371/journal.pgen.1004039)
Supplement: Table S1 — Q-PCR primers used in the expression analysis. (DOCX) [file pgen.1004039.s009.docx]

**Table S1. Q-PCR primers used in expression analysis.**

| Gene target | Forward primer | Reverse primer |
| --- | --- | --- |
| Mkrn3 | ACAGGTGTGCATACCCCCA | GCAGGCCCTTCTATGAGCTTC |
| Magel2 | ATCCAGTCTCAAGTCATAAGGGC | CTGCCATGTCAAAGGCGTT |
| Ndn | GAGGTCCCCGACTGTGAGAT | TGCAGGATTTTAGGGTCAACATC |
| Snrpn U1-3 | AAAGGAGCCTGACACATCCA | CCTTGAATTCCACCACCTTG |
| Snrpn | TGCTACGTGGGGAGAACTTG | CCTGGGGAATAGGTACACCTG |
| Snord116 | GGATCTATGATGATTCCCAG | GGACCTCAGTTCCGATGA |
| Ipw | GATGCATTCCTTTTCCTTCA | TGGTAGAAGAAATGGCACCATC |
| Ube3a | GCACCTGTTGGAGGACTAGG | GTGATGGCCTTCAACAATCTC |
| Ube3a-ATS 5’ | ACAGAACAATAGGTCACCAGGTT | AAGCAAGACTGTTCACCTCAT |
| Ube3a-ATS 3’ | CCAATGACTCATGATTGTCCTG | GTGATGGCCTTCAACAATCTC |
| Atp10a | GTTGTGCCACATCGAGACTG | GTGAACGTCAGAGGGTTGAAT |
| Gapdh | TGACCACAGTCCATGCCATC | GACGGACACATTGGGGGTAG |
