# Supplementary material for: Truncation of Ube3a-ATS Unsilences Paternal Ube3a and Ameliorates Behavioral Defects in the Angelman Syndrome Mouse Model
Source: PLoS Genet. 2013 Dec 26;9(12):e1004039. doi: 10.1371/journal.pgen.1004039 (PMC3873245; doi:10.1371/journal.pgen.1004039)
Supplement: Table S2 — RT and q-PCR primers used in analyzing Ube3a transcription initiation. (DOCX) [file pgen.1004039.s010.docx]

**Table S2. RT and q-PCR primers used in analyzing Ube3a transcription initiation.**

| Locus | RT primer | q-PCR forward primer | q-PCR reverse primer |
| --- | --- | --- | --- |
| Ube3a-int1 | **GGCCGTCATGGTGGCGAATA**AACTGGGAGACATGAGGTTAGA | GAAGAGTCTGGGAGAACAGTAAA | GGCCGTCATGGTGGCGAATA |
| Ube3a-int3 | **GGCCGTCATGGTGGCGAATA**TTCAGGACATGCTCCTAATT | AGTCTTGGGTTGGTAGTGGA | GGCCGTCATGGTGGCGAATA |
| Ube3a-int4.2 | **GGCCGTCATGGTGGCGAATA**ACCTATGACAGGCATTATCT | TACTGACTGTTCCCTCTTGA | GGCCGTCATGGTGGCGAATA |
| Ube3a-int4.4 | **GGCCGTCATGGTGGCGAATA**CTAACACCCACAGTTTATTC | CAGTTCTCATTCAGCCATTT | GGCCGTCATGGTGGCGAATA |
| Ube3a-int6.2 | **GGCCGTCATGGTGGCGAATA**GGAGAATAAAGACGTGGAAAG | GTGGGAAAGGGTTAGAAATAG | GGCCGTCATGGTGGCGAATA |
| Ube3a-int12.3 | **GGCCGTCATGGTGGCGAATA**AAGGGAAAATATCCATTCCACTA | ATTCCATACACGCAAGCAGAG | GGCCGTCATGGTGGCGAATA |
| Ube3a-ex1-4 | N_6_ | TTCGTCTTTGCCAGCACCTCG | GTCCTCAGACTGGGATTCTCC |
| Ube3a-ex4-6 | N_6_ | GAATCCCAGTCTGAGGACAT | TGTGCTGTTTGACTTTCCGA |
| Ube3a-ex12-13 | N_6_ | GCACCTGTTGGAGGACTAGG | GTGATGGCCTTCAACAATCTC |

Sequences in bold font indicate tag sequences.
