# Supplementary material for: Truncation of Ube3a-ATS Unsilences Paternal Ube3a and Ameliorates Behavioral Defects in the Angelman Syndrome Mouse Model
Source: PLoS Genet. 2013 Dec 26;9(12):e1004039. doi: 10.1371/journal.pgen.1004039 (PMC3873245; doi:10.1371/journal.pgen.1004039)
Supplement: Text S1 — Materials and methods. (DOCX) [file pgen.1004039.s011.docx]

**Materials and Methods**

*Open field assay:* Each mouse is placed in the center of a clear Plexiglas (40x40x30 cm) open-field arena (Versamax Animal Activity Monitor, AccuScan Instruments, Columbus, OH) and allowed 30 min to explore. Overhead lighting is approximately 800 lux inside the field, and the white noise is at approximately 60 dB. Mouse activity, such as total distance traveled, time of movement, center distance traveled, etc., is recorded and quantified.

*Marble burying test:* Each mouse is placed in a standard mouse cage containing 20 small (1.5-2 cm) clean black marbles on top of 4 inches of corn cob bedding, forming 4 rows of 5 columns. After a period of 30 min exploration, the mouse is removed from the cage and the number of marbles that are buried at least 50% is recorded.

*Wire Hang Test:* A single 1-3mm wire of 1 m long is suspended approximately 0.4 m above a plastic-covered foam pad. Each mouse is placed in the middle of the wire by allowing it to grasp only with its forepaws. Latency to fall is measured with a 60-sec cutoff time.

*Dowel test:* A wooden dowel with 1 m length and 8mm diameter is suspended approximately 0.4 m above a plastic-covered foam pad. Each mouse is gently placed in the middle of the beam. Latency to fall off the dowel is measured with a 120-sec cutoff time.

*Accelerating rotarod:* The test is performed with a rotating rod system that rotates from 4 to 40 rpm within 5 min (model 7650 Rota-rod, Ugo Basile, Collegeville, PA). Mice are placed on the rotating rod and the time of mice remaining on the rod until falling off or losing balance (mice not walking on the rod for two consecutive turns) is recorded. For two consecutive days, four trials were performed per day with at least 30 min interval between trials.

*Fear conditioning:* The experimenter was blind to the genotype. Fear conditioning was performed as previously described (Huang et al., Nat Neurosci, 2013; Zhu et al., Cell, 2011). Briefly, mice were first handled for 2-3 min for 3 days. On the training day, after 2 min in the conditioning chamber, mice received two pairings of a tone (2800 Hz, 85 db, 30 s) with a co-terminating foot-shock (0.7 mA, 2 s), after which they remained in the chamber for one additional min and then were returned to their home cages. At 24 hr after training, mice were returned to the conditioning chamber for 5 min and tested for “freezing” (immobility with the exception of respiration) in response to the training context. Freezing behavior was analyzed with FreezeView (Actimetrics, Wilmette, IL). The percent of time spent freezing was taken as an index of learning and memory.
